# Supplementary material for: Systemic Inflammation Predicts Alzheimer Pathology in Community Samples without Dementia
Source: Biomedicines. 2022 May 26;10(6):1240. doi: 10.3390/biomedicines10061240 (PMC9219863; doi:10.3390/biomedicines10061240)
Supplement: Supplementary file 1 [file biomedicines-10-01240-s001.zip › biomedicines-1563994-supplementary.pdf]

SUPPLEMENTARY MATERIAL

Methods

Sample collection and processing

Blood draws contributing to these analyses were conducted at the fourth assessment wave of the PATH study. Samples were collected after a fast of at least 8 hours, stored at -80C aliquoted in 1ml vials. Immediately prior to analysis samples were thawed, diluted 1:2 (except total tau: 1:1), and were all processed with the same pipeline at the same time at the ANU Phenomics Facility. All markers were assessed in duplicates in plasma as recommended by assay manufacturers (Mesoscale, Rockville, USA). Controls included in kits were compared to the manufacturer's guide range, and the robustness of the data was confirmed by the manufacturer.

Inflammatory cytokines and OS markers

TNFα, TNF-R1, TNF-R2, IL1β, IL4, IL6, IL8, IL10 were measured on the Mesoscale platform with the human high sensitivity cytokine and pro-inflammatory panel 1 assays (Rockville, MD, USA). Other measures were assessed on ELISA assays as follows: NO (ELISA OxiSelect™, Jomar Bioscience, STA-802), Neo (Human BioAssay, Jomar Biosciences, #3410), TAC (Abcam, #Ab65329), MDA (ELISA OxiSelect™, Jomar Bioscience, STA-832), GUA (Oxidative Damage EIA kit, Sapphire Biosciences, #190-58932). The mean coefficient of variation across all assays was 16.7% (range 0.91%-49.3%). IL1α was also assayed but did not pass quality control and therefore was not included in analyses.

AD Biomarkers

Amyloid beta 38, 40, and 42 as well as total tau were measured on the Mesoscale platform with the human high sensitivity V-PLEX Plus Aβ Peptide Panel 1 assay (Rockville, MD, USA). The mean coefficient of variation across all assays was 9.84% (range 2.33%-14.30%).

Principal component analysis

Pearson bivariate correlations are presented in **Figure S1** and indicate that none of the markers were exclusively positively or negatively associated with other measures. TNF-R1 and TNF-R2 were the most highly correlated measures. Notably, the shared variance between pairs of markers was on average relatively low and ranged from 0.04% to 23% (with one exception TNF-R1 & TNF-R2 at 69% in the 40s and 74% in the 60s). NO, GUA and TAC shared least variance with other markers while IL6 and IL10 shared most variance in the 40s, and IL4 and IL8 in the 60s.

Table S1. Factor loadings of OS/inflammatory markers on each principal component in the 40s.

```
##                   PC1          PC2         PC3         PC4         PC5
## il1b_log  0.35549276 -0.138709548  0.04676871 -0.23414727  0.01028207
## il4_log   0.36136000 -0.229231177 -0.06113265 -0.08746830  0.14671930
## il6_log   0.37498420 -0.049793262  0.05454829 -0.12286565 -0.13433684
## il8_log   0.35744112 -0.107003218 -0.14312049 -0.19225157  0.28112500
## il10_log  0.36447331 -0.232806749  0.09994211  0.05564043 -0.19187304
## no_imp   -0.04861748  0.188235556  0.40989792 -0.37237492  0.53782961
## tac      -0.07203701  0.184570053 -0.65888968  0.09630026  0.51465976
## neo_log   0.19761446  0.213667769 -0.14300767  0.47973896 -0.07510971
## mda_imp   0.06058994  0.008262575  0.50788114  0.42733235  0.46421450
## gua_imp  -0.04129848  0.320108196 -0.11540311 -0.54721610 -0.08317370
## tnfa_imp  0.43965970  0.052857229 -0.19526967  0.09482321  0.15827884
## tnf1_imp  0.25247324  0.545211454  0.15836976 -0.02778062 -0.15404910
## tnf2_imp  0.18111101  0.585102641  0.05960371  0.11164219 -0.11619679
##                   PC6          PC7         PC8         PC9        PC10
## il1b_log -0.19632740 -0.158452320  0.32319449  0.68204011  0.25358989
## il4_log  -0.13559926  0.076574275  0.03376103 -0.64432790  0.33103471
## il6_log  -0.03913460  0.132260642 -0.60452098  0.12274934 -0.55327787
## il8_log   0.10894890  0.017134561  0.48587513 -0.15593723 -0.43708454
## il10_log  0.11508256 -0.037916439 -0.35830247  0.02271090  0.47808611
## no_imp    0.55466428  0.006351416 -0.16990557  0.05089197  0.14084622
## tac      -0.15673404  0.138640806 -0.27649450  0.18751594  0.15076803
## neo_log   0.44344687 -0.600236039  0.04192752 -0.02635498 -0.04849117
## mda_imp  -0.51365820 -0.223056007 -0.06977799 -0.01487929 -0.09282621
## gua_imp  -0.32025708 -0.617056558 -0.16033735 -0.19007991  0.01981118
## tnfa_imp  0.08061831 -0.015421973 -0.06459604  0.04921917 -0.03227945
## tnf1_imp -0.10997536  0.222252246  0.10491185 -0.04363276 -0.04070796
## tnf2_imp -0.05056316  0.300189148  0.10804348 -0.02526520  0.20917510
##                  PC11         PC12          PC13
## il1b_log -0.26923098 -0.163591369 -0.0206473206
## il4_log  -0.44562855 -0.187713606 -0.0018334710
## il6_log  -0.19225996 -0.248721290 -0.1277091596
## il8_log   0.47766529 -0.178926370 -0.0231208948
## il10_log  0.60742926 -0.063733849  0.1337308520
## no_imp   -0.08211973 -0.023052975 -0.0004365039
## tac       0.07031079 -0.218174709  0.1476057537
## neo_log  -0.17440170 -0.255262948  0.0417735601
## mda_imp   0.11119901 -0.004015447 -0.0228509045
## gua_imp   0.13524930  0.075044657 -0.0816430905
## tnfa_imp -0.08885865  0.834961579 -0.1109080562
## tnf1_imp -0.04015933  0.016498067  0.7151746743
## tnf2_imp  0.11208085 -0.153918392 -0.6405828060
```

Table S2. Factor loadings of OS/inflammatory markers on each principal component in the 60s.

```
##                  PC1         PC2         PC3         PC4         PC5
## il1b_log -0.19692889  0.39867568 -0.14135699  0.05991506 -0.18207266
## il4_log  -0.05448955  0.50053845  0.03060614 -0.24308831 -0.01845252
## il6_log  -0.31414439  0.08973366 -0.08929462  0.10749212 -0.23320683
## il8_log  -0.25607262  0.35906246  0.01557060 -0.01243623  0.45227926
## il10_log -0.34567899  0.25999833 -0.12008494  0.04144134  0.13887540
## no_imp   -0.10155185 -0.28157324 -0.13387649 -0.40815245  0.65377685
## tac      -0.02924091 -0.26844423 -0.58939831  0.30633397  0.16325693
## neo_log  -0.32960398 -0.06834356 -0.15060838 -0.17653678 -0.26698418
## mda      -0.07018977  0.06253222  0.56116967  0.57228747  0.33878935
## gua_imp  -0.15556666 -0.14144232  0.43138690 -0.52766194 -0.06739159
## tnfa_imp -0.40573594  0.07326627 -0.14961912 -0.01879217  0.12003951
## tnf1_imp -0.40687387 -0.31488760  0.16300242  0.10310763 -0.14526357
## tnf2_imp -0.44203379 -0.31857002  0.13596769  0.12398828 -0.08995743
##                   PC6          PC7         PC8         PC9        PC10
## il1b_log  0.384979513 -0.455846206 -0.09771580 -0.55859705  0.11741294
## il4_log   0.361307258  0.390492846 -0.43402111  0.27138691  0.03544971
## il6_log   0.271947841 -0.076043998  0.70864598  0.30642547 -0.16052117
## il8_log  -0.249104580  0.003114931  0.06023093 -0.24877359 -0.55105125
## il10_log -0.051684445 -0.061214224  0.03563744  0.43637625  0.43427122
## no_imp    0.321779212  0.040944776  0.21175175 -0.17356512  0.30586484
## tac       0.147544160 -0.347277569 -0.38697509  0.27034151 -0.19396950
## neo_log  -0.470125412 -0.073390549 -0.07332567 -0.20408895  0.41654883
## mda      -0.004675704 -0.199103084 -0.06777691  0.05543680  0.27358122
## gua_imp   0.020055487 -0.575955925 -0.16934486  0.28148155 -0.21421411
## tnfa_imp -0.374605715  0.097787744 -0.07159250  0.09676866 -0.13287777
## tnf1_imp  0.286292851  0.273902802 -0.08709458 -0.16209967 -0.14927881
## tnf2_imp  0.116282718  0.215785054 -0.22147880 -0.07281565 -0.03969980
##                 PC11        PC12          PC13
## il1b_log -0.14812378 -0.17805669  0.0232374447
## il4_log   0.37307672 -0.02503612 -0.0007016553
## il6_log   0.32982281 -0.04285609  0.0845391445
## il8_log   0.06232242  0.40190357  0.0624534143
## il10_log -0.53758354  0.31597326 -0.0440836766
## no_imp    0.12283485 -0.09765097  0.0104317988
## tac       0.21060126  0.09821966 -0.0486782663
## neo_log   0.49990455  0.24807407 -0.0563067302
## mda       0.31003046 -0.10518567 -0.0678240681
## gua_imp  -0.01360835 -0.00118391 -0.0248678038
## tnfa_imp -0.08356285 -0.76082260 -0.1580824603
## tnf1_imp -0.11239557  0.18362915 -0.6461518967
## tnf2_imp -0.10929062  0.01034899  0.7301364042
```

Table S3. Association between C-Reactive Protein (CRP) and inflammatory states identified in the PCA. Note that CRP has been log transformed to account for extreme kurtosis. Results were consistent regardless of covariates, coefficients reported here come from models controlling for gender, age, education, hypertensive status, type 2 diabetes status, Goldberg depression inventory, BMI, and smoking (pack years).

Cohort	DV	Estimate	Std. Error	t value	Pr(>|t|)
MA	PC1	0.173210	0.196250	0.883	0.3785
MA	PC2	0.043083	0.141687	0.304	0.7614
MA	PC3	-0.146636	0.117845	-1.244	0.2149
MA	PC4	-0.051431	0.111152	-0.463	0.6441

Cohort	DV	Estimate	Std. Error	t value	Pr(>|t|)
OA	PC1	-0.071890	0.163814	-0.439	0.6613
OA	PC2	-0.002609	0.117017	-0.022	0.9822
OA	PC3	-0.158010	0.096304	-1.641	0.1030
OA	PC4	-0.115707	0.092966	-1.245	0.2148

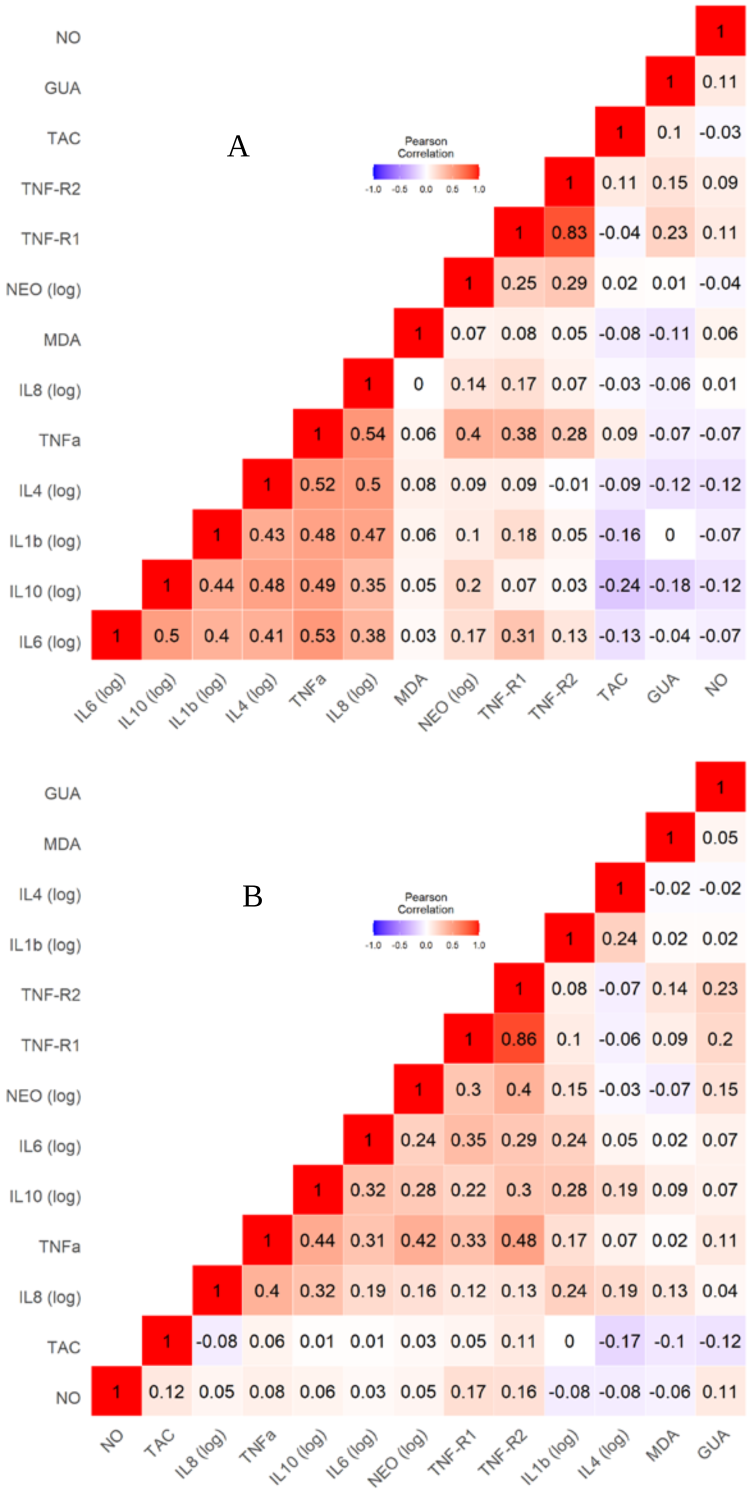

Figure S1. Pearson bivariate correlations between the inflammatory markers in (A) the 40s, and (B) the 60s.

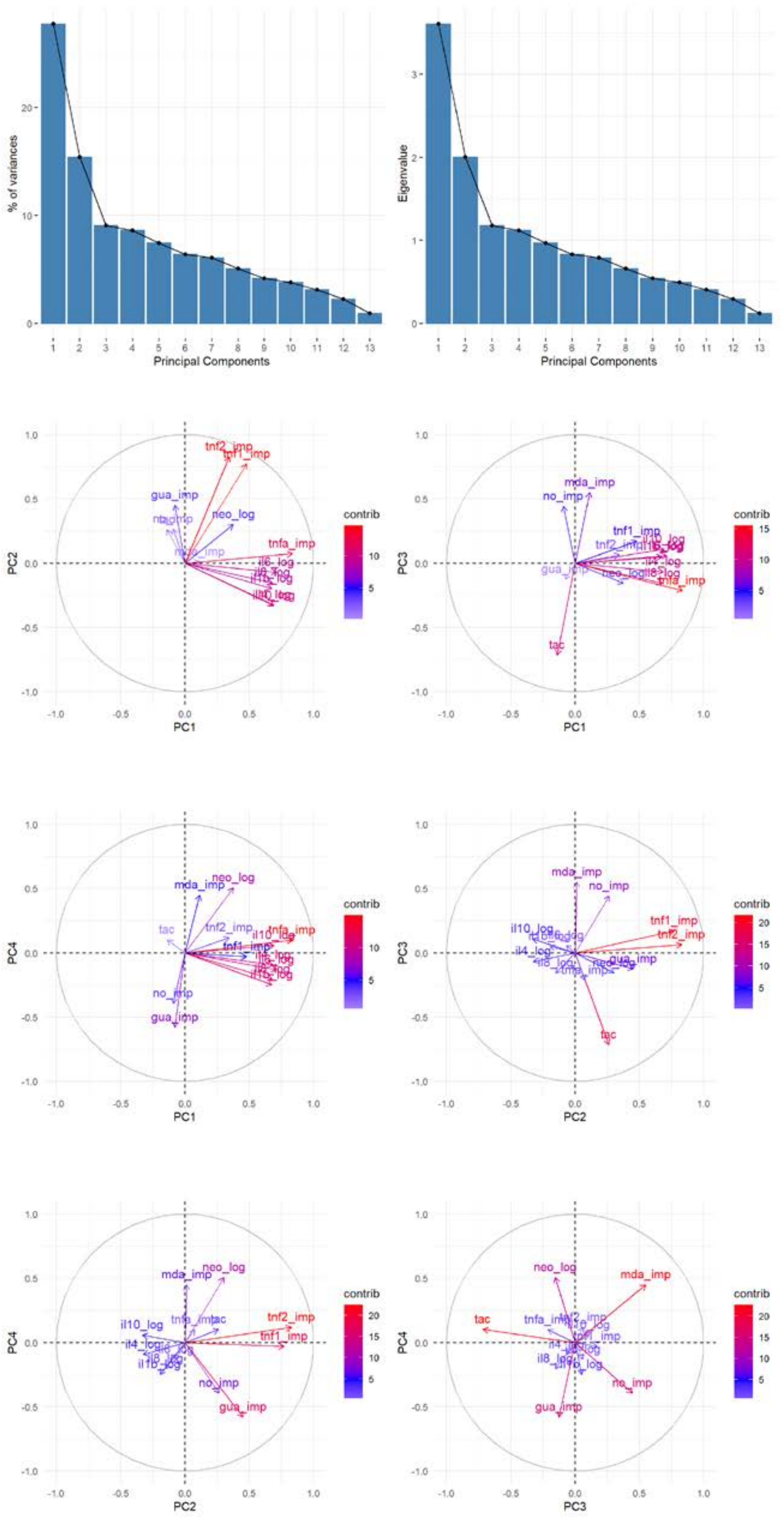

Figure S2. Proportion of variance in OS/inflammatory markers explained by principal components (top left) and their eigen values (top right) in the 40s. Spatial depiction in bivariate space of the markers contributing to different principal components (lower figure).

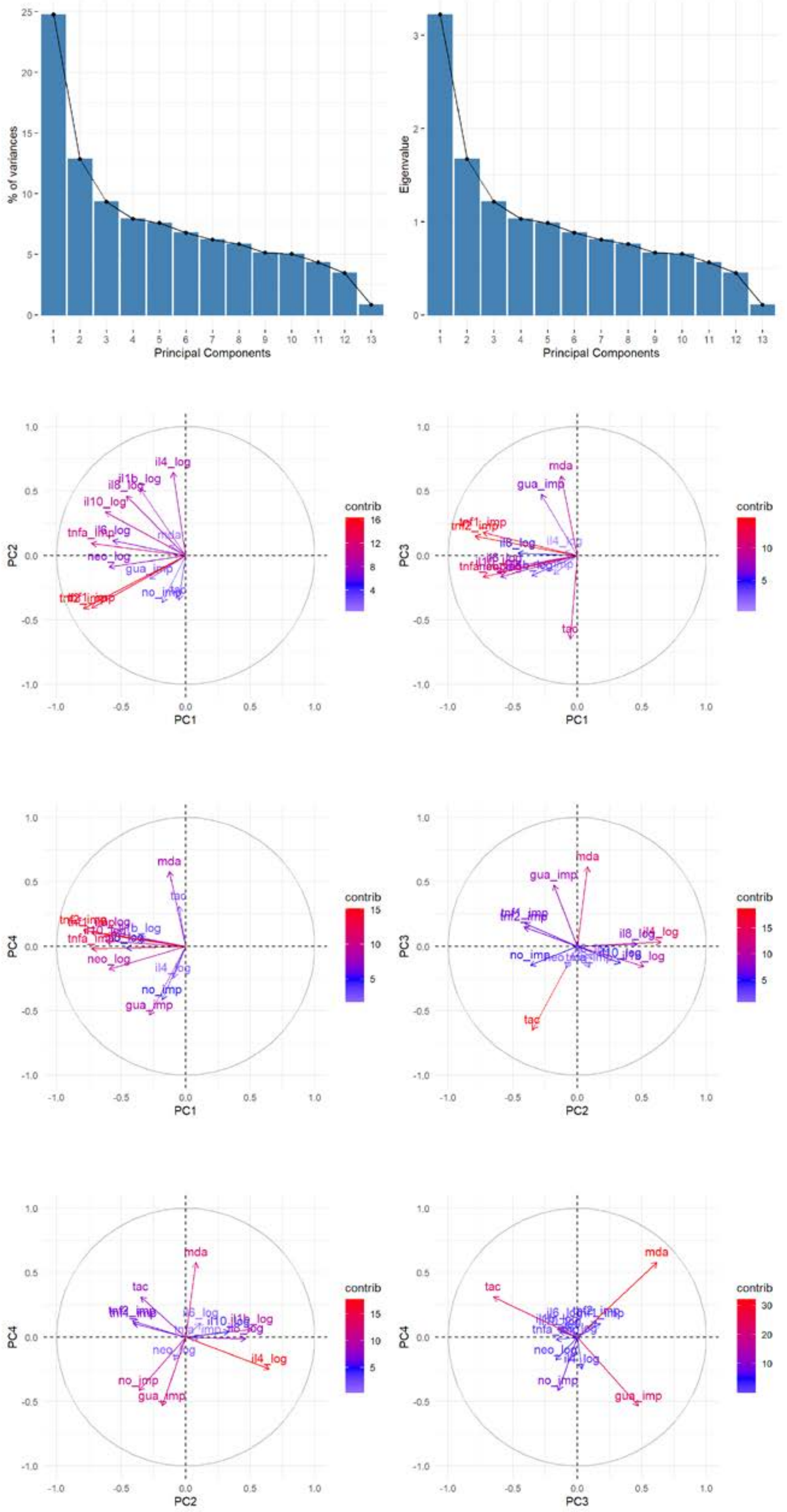

Figure S3. Proportion of variance in OS/inflammatory markers explained by principal components (top left) and their eigen values (top right) in the 60s. Spatial depiction in bivariate space of the markers contributing to different principal components (lower figure).

A

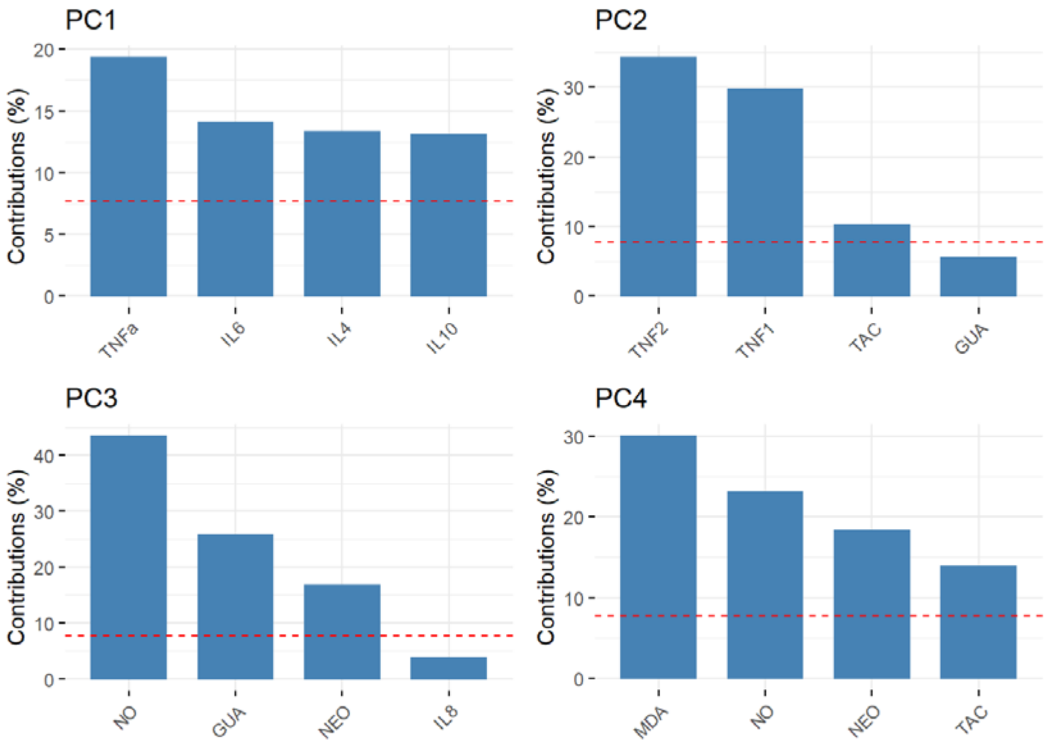

B

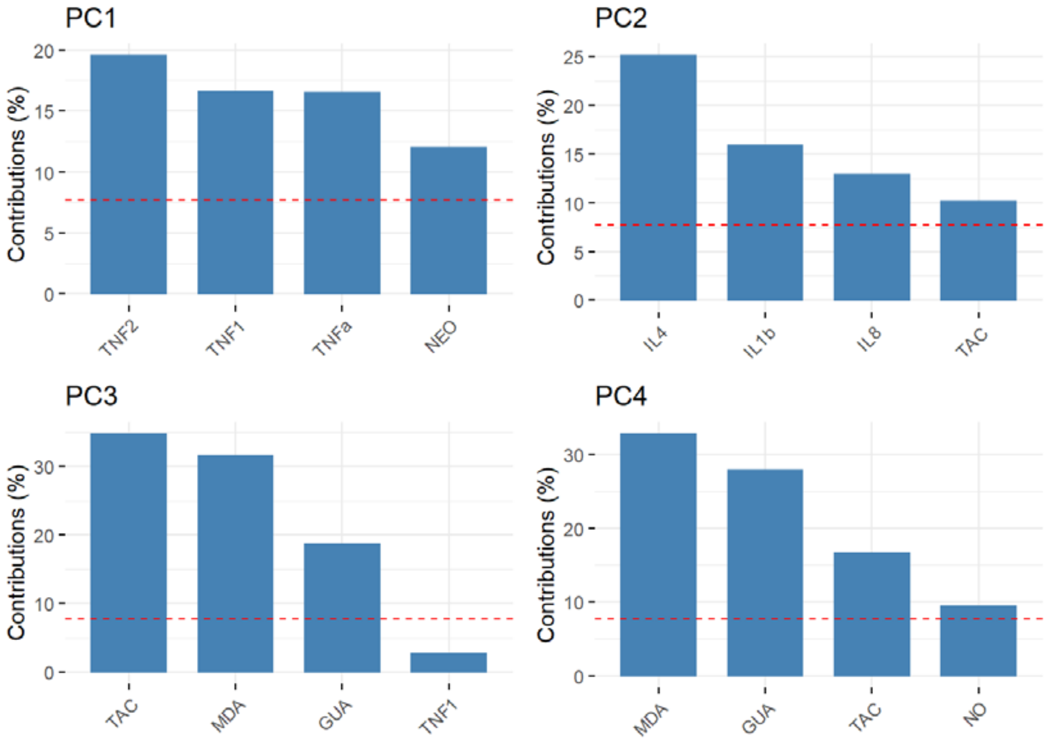

Figure S4. Main oxidative stress and inflammatory markers' contribution to each of the four significant principal components (PC1-PC4) identified in the PCA analysis for A) the 40s and B) the 60s. Red line represents the significance threshold.

A

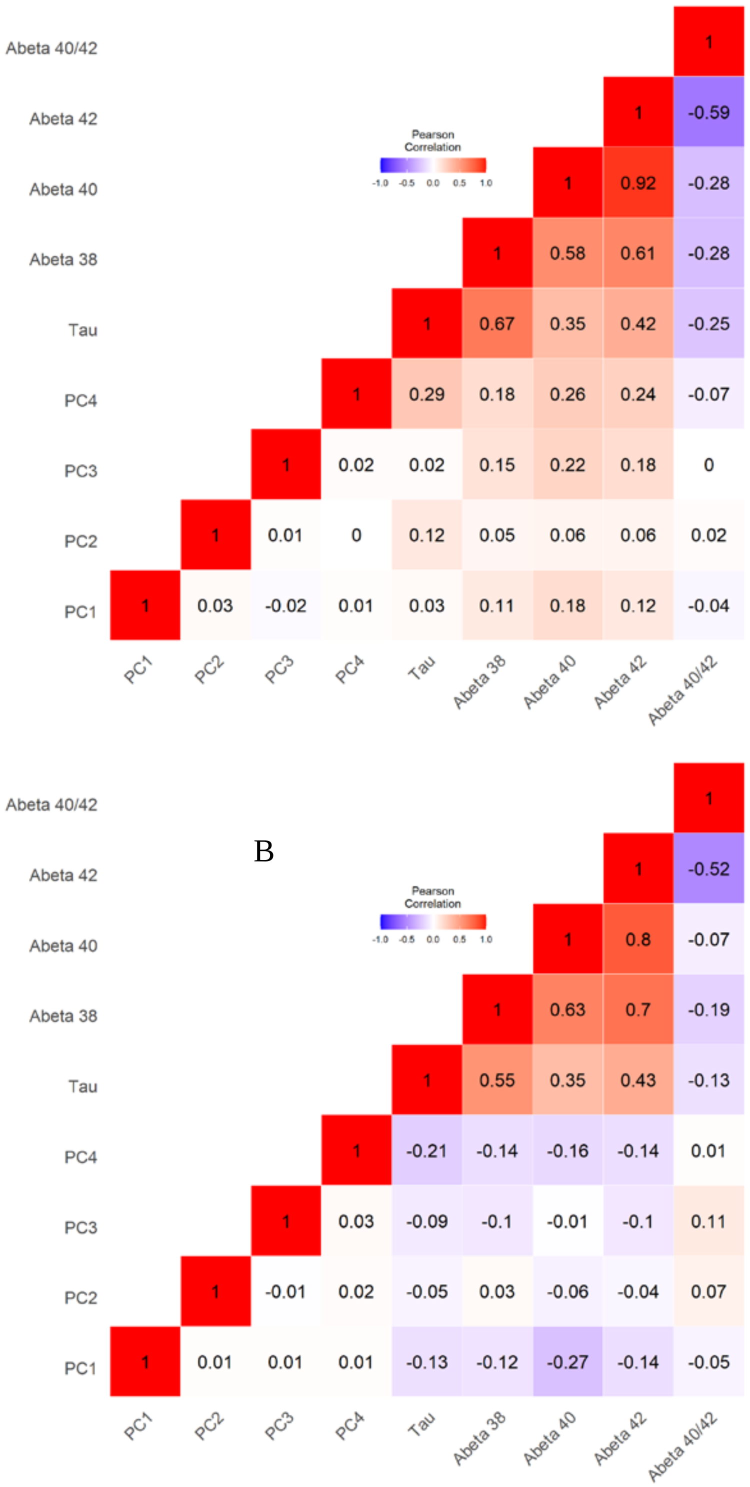

Figure S5. Bivariate Pearson correlations between the principal components (PC1-PC4) and AD biomarkers (Aβ 38/40/42, Tau) in (A) the 40s, and (B) the 60s.
